# Use of combined treatment of 3rd-generation cephalosporin, azithromycin and antiviral agents on moderate SARs-CoV-2 patients in South Korea: A retrospective cohort study

**DOI:** 10.1371/journal.pone.0267645

**Published:** 2022-05-04

**Authors:** Wooyoung Hong, Yu-Kyung Park, Bong-Ok Kim, Sung Kyu Park, Jiin Shin, Soon-Pyo Jang, Hea-Woon Park, Wonjong Yang, Joonyoung Jang, Soon-Woo Jang, Tae-Ho Hwang

**Affiliations:** 1 Department of Chemistry and Chemical Biology, Harvard University, Cambridge, MA, United States of America; 2 Korea Workers’ Compensation & Welfare Services Daegu Hospital, Buk-gu, Daegu, Republic of Korea; 3 School of Medicine, Seoul National University, Seoul, Republic of Korea; 4 Department of Oral and Maxillofacial Surgery, Division of Oral Pathobiological Science, Graduate School of Dental Medicine, Hokkaido University, Hokkaido, Japan; 5 Director of Bukha Public Health Center, Jangseong, Republic of Korea; 6 Pusan University, School of Medicine, Yangsan, Republic of Korea; 7 Department of Pharmacology, School of Medicine, Pusan National University, Yangsan, Republic of Korea; 8 Gene and Cell Therapy Research Center for Vessel-associated Diseases, School of Medicine, Pusan National University, Yangsan, Republic of Korea; University of Sassari, ITALY

## Abstract

**Objectives:**

To assess efficacy and safety of the combined treatment of antibiotics (3rd-generation cephalosporin and azithromycin) and antiviral agents (lopinavir/ritonavir or hydroxychloroquine) on moderate COVID-19 patients in South Korea.

**Methods:**

A retrospective cohort study of the 358 laboratory-confirmed SARS-CoV-2 (COVID-19) patients was conducted. 299 patients met inclusion criteria for analysis. Propensity score matching (PSM) and Cox regression method were used to control and adjust for confounding factors. Mild to moderate COVID-19 patients were managed with either CA/LoP (cephalosporin, azithromycin, and lopinavir/ritonavir) (n = 57), CA/HQ (cephalosporin, azithromycin, and hydroxychloroquine) (n = 25) or standard supportive care (n = 217). We analyzed the association between treatment group and standard supportive group in terms of three endpoints: time to symptom resolution, time to viral clearance, and hospital stay duration. Using propensity-score matching analysis, three rounds of propensity-matching analysis were performed to balance baseline characteristics among three cohorts.

**Results:**

Kaplan-Meier curves fitted using propensity score-matched data revealed no significant differences on time to symptom resolution, time to viral clearance, hospital stay duration among the three treatment arms (CA/LoP vs Standard, log-rank p-value = 0.2, 0.58, and 0.74 respectively for the three endpoints) (CA/HQ vs Standard, log-rank p-value = 0.46, 0.99, and 0.75 respectively). Similarly, Cox regression analysis on matched cohorts of CA/LoP and standard supportive group showed that hazard ratios of time to symptom resolution (HR: 1.447 [95%-CI: 0.813–2.577]), time to viral clearance(HR: 0.861, [95%-CI: 0.485–1.527]), and hospital stay duration (HR: 0.902, [95%-CI: 0.510–1.595]) were not significant. For CA/HQ and standard supportive group, hazard ratios of the three endpoints all showed no statistical significance (HR: 1.331 [95%-CI:0.631–2.809], 1.005 [95%-CI:0.480–2.105], and 0.887, [95%-CI:0.422–1.862] respectively). No severe adverse event or death was observed in all groups.

**Conclusions:**

Combined treatment of 3rd cephalosporin, azithromycin and either low-dose lopinavir/ritonavir or hydroxychloroquine was not associated with better clinical outcomes in terms of time to symptom resolution, time to viral clearance, and hospital stay duration compared to standard supportive treatment alone. Microbiological evidence should be closely monitored when treating SARS-CoV-2 patients with antibiotics to prevent indiscreet administration of empirical antimicrobial treatments.

## 1. Introduction

As of June 27^th^ 2020, over 219,000,000 confirmed cases and over 4,500,000 deaths due to Coronavirus Disease 2019 (COVID-19) were reported by the World Health Organization (WHO) [1]. The causative virus of this pandemic, SARS-CoV-2, presented an unprecedented challenge to healthcare systems worldwide, but no definitive treatment protocol exists due to lack of clear understanding of its pathogenesis and its nature [2]. In South Korea, Daegu city had the first large outbreak of SARS-CoV-2 outside China [3]. The number of early SARS-CoV-2 infections in Daegu increased rapidly, but numerous hospitals in South Korea were not well prepared to deal with this unexpected catastrophic event. Accordingly, hospitals in South Korea had to treat SARS-CoV-2 patients with management methods such as standard supportive care alone or repositioning of old drugs which include antibiotics such as 3^rd^ cephalosporin, azithromycin, etc, or antiviral agents such as hydroxychloroquine (HQ), lopinavir/ritonavir (LoP/R), etc [3–5]. Also, these repositioned drugs were recommended by some non-randomized studies, and Emergency Use Authorization of FDA suggested using antibacterial, antiviral, antiinflammatory drugs to treat SARS-CoV-2 [5–7].

At the early stage of SARS-CoV-2 infection, symptoms of mild fever and shortness of breath were observed [8], but this virus quickly replicates in the respiratory tract and begins to infect the alveoli. This pathogenesis causes host immune to cause hyper-inflammatory responses which result in altering microvascular permeability to induce tissue edema [9], and the inflamed fluid-filled alveolar tissue is an ideal habitat for bacterial growth for pathogens including *P*.*aeruginosa and S*.*aureus* [10]. This secondary bacterial coinfection had been a real concern to SARS-CoV-2 patients, as a high mortality rate of 15.2% was observed for patients with pneumonia caused by the secondary bacterial infections following SARS-CoV-2 infections. Correspondingly, antimicrobial agents have been used as treatment and prophylactic measures to prevent the secondary infections and worsen the disease [11], as recommended by the Korean Society of Infectious Disease [12] and other literature [13–17].

In South Korea, the third generation cephalosporin has been widely used to treat bacterial pneumonia. Depending on the traits of suspected pathogens and their anticipated susceptibility in South Korea, this drug has been recommended as an empiric antimicrobial therapy to prevent and manage respiratory infections [18]. While cephalosporin has been widely used to prevent the secondary bacterial infection, ceftaroline fosamil which is a member of cephalosporin antibiotics was suggested as a potent inhibitor of SARS-CoV-2 main protease responsible for processing the polyprotein translated from viral RNA in preclinical study [19]. Also, macrolides were known to be effective for viruses as well as bacteria. Specifically, azithromycin was proven to be effective for rhinovirus, respiratory syncytial virus, influenza virus, zika virus, and ebola virus *in vitro* [20–22].

Among antiviral agents, hydroxychloroquine, which is generally used to treat malaria and rheumatic diseases, was noted for its anti-SARS-CoV activity *in vitro* [23, 24] as well as its well-known safety and predictable adverse effects [25]. Gautret P et al. reported that hydroxychloroquine was effective at reducing the viral load in COVID-19 patients in the French population [6, 26]. Lopinavir, which is a HIV-1 protease inhibitor, combined with ritonavir to increase serum concentration through CYP450 inhibition, showed an inhibitory effect on the replication of Severe Acute Respiratory Syndrome Coronavirus (SARS-CoV), SARS-CoV-2 *in vitro* [27, 28]. X-T. Ye et al. reported that treatment with lopinavir/ritonavir (LoP/R) combined with adjuvant drugs showed an evident therapeutic effect in lowering the body temperature and negatively converting nCoV-RNA with no side effects in SARS-CoV-2 patients [5]. Given the urgency of COVID-19 outbreak, the Food and Drug Administration (FDA) issued Emergency Use Authorization on March 30, 2020, based on prior studies. It allowed the use of hydroxychloroquine and LoP/R as a treatment for SARS-CoV-2 patients not enrolled in clinical trials.

The mortality and severity of SARS-CoV-2 patients vary across regions [29]. Based on a study from China, roughly 80% of SARS-CoV-2 patients in the Northeast Asian countries including South Korea showed non-severe symptoms [30]. Likewise, 80.3% of SARS-CoV-2 patients in Keimyung University Dongsan Hospital, the biggest COVID-19 healthcare center in Daegu, where the first large outbreak occurred in South Korea, mostly had mild or asymptomatic cases [31]. Thus, it is relevant to investigate the efficacy of the treatments addressed to these patients with a non-severe status of the disease.

While South Korea has been relatively successful with its epidemiologic control strategies and thus the pandemic containment [32], pharmacological management and treatment of its infected citizens have not been reported as extensively. Also, since some clinical trials related to the pharmacological treatments of SARS-CoV-2 could not get completed because of frequent updates in management guidelines and controversial results in various studies, no definitive treatment protocol to this new disease is yet established. Yet, some other clinical trials related to the new drugs for SARS-CoV-2 are still underway [33]. The combination of antiviral agents and antibiotics was widely used to treat SARS-CoV-2 patients globally in practice, and some existing studies reported efficacy and safety of antiviral agents in SARS-CoV-2 [34, 35], but studies on those of antibiotics with solid clinical results are still lacking despite their wide empirical use [36]. Thus, here we present our experience on SARS-CoV-2 management with pharmacological therapy based on clinical research. This study aims to investigate treatment responses and safety from South Korean SARS-CoV-2 patients who received either treatments with antibiotics and antiviral agents or supportive treatments.

## 2. Methods

### Study design

**Fig 1:**
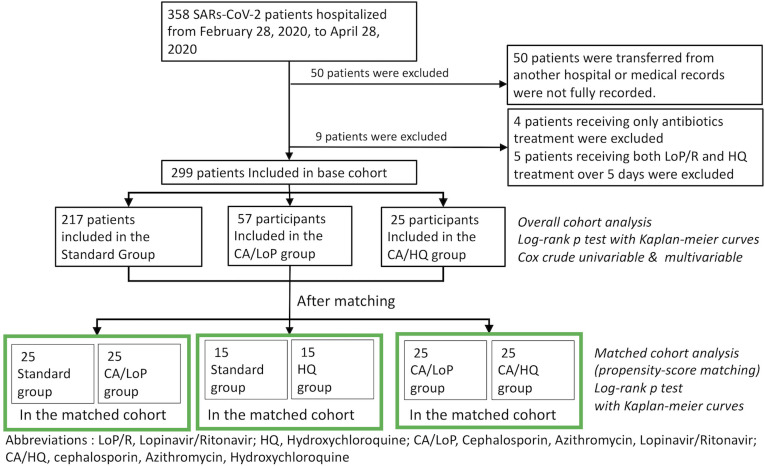
Flowchart of the study. Abbreviations: LoP/R, Lopinavir/Ritonavir; HQ, Hydroxychloroquine; CA/LoP, Cephalosporin, Azithromycin, Lopinavir/Ritonavir; CA/HQ, cephalosporin, Azithromycin, Hydroxychloroquine.

This study is an observational retrospective cohort study of 358 patients with laboratory-confirmed SARS-CoV-2 infection hospitalized in Korea Worker’s Compensation & Welfare Service Daegu Hospital, a quarantine facility for mild-moderate SARS-CoV-2 patients. Before admission, all patients were diagnosed with SARS-CoV-2 by real-time reverse-transcriptase polymerase chain reaction (RT-PCR) according to the WHO protocol [37]. After being diagnosed positive, confirmed patients were hospitalized. Study period was from February 28, 2020, to April 28, 2020, which was the operating period of the quarantine facility. During this period, 358 patients were hospitalized in this quarantine facility. Among 358 COVID-19 patients, 299 patients remained appropriate for full analysis as some patients were removed as described below. First, 50 patients who were transferred from other hospitals to our hospital were excluded from the analysis, as their medical records were not fully available. Second, 5 patients who received both lopinavir/ritonavir and hydroxychloroquine for more than 5 days and 4 patients who received only the antibiotics were also excluded. The experimental groups consisted only with the patients who received at least three days of antiviral agents (HQ or LoP/R) with antibiotics (Cefixime and Azithromycin) or any duration of supportive standard treatment. 217 patients were in standard group (patients only received standard Supportive treatment) and 82 patients were in the treatment group. Within the treatment group, 57 patients were treated with cefixime, azithromycin, and lopinavir/ritonavir (CA/LoP group), and 25 patients were treated with cefixime, azithromycin, hydroxychloroquine group (CA/HQ group). Treatment group was defined as CA/LoP group and CA/HQ group. **Fig 1** is the flowchart of the study **(Fig 1)**.

The authors reviewed the electronic medical records of patients and collected epidemiological, clinical, historical, laboratory, and treatment outcomes data. Patient confidentiality was protected by de-identifying patient information. The anonymous electronic data was stored in a locked, password-protected computer. The ethics committee of Pusan National University Yangsan Hospital approved this study and granted a waiver of informed consent from study participants in accordance with Korean legislation on non-interventional studies (IRB No.: 05-2020-082). The research data that support the findings of this study are available from the corresponding author upon reasonable request.

### Laboratory tests

The SARS-CoV-2 nucleic acid amplification test (NAAT, Real-time RT-PCR) was performed by using PowerChek^TM^ 2019-nCoV Real-time PCR kit (Kogene Biotech, Seoul, Korea) in Bio-Rad CFX96 Deep Well real-time PCR detection systems (Bio-rad, Hercules, CA, USA), after viral RNA extraction by using NX-48 viral nucleic acid extraction kit (Genolution, Seoul, Korea) in conjunction with Nextractor NX-48 (Genolution). This assay targets the two genes (E: for *Sarbecovirus* screening, RdRp for confirmation of SARS-CoV-2) as suggested by the KCDC and WHO [37, 38]. The result of the E and RdRp target means the presence of SARS-CoV-2. A positive test result was defined when a well-defined exponential fluorescence curve crossed the threshold ≤ 35 cycles for the E and RdRp genes respectively. Limit of the detection point for SARS-CoV-2 is less than 10 copies/uL according to the manufacturer’s insert. We collected data of C_t_ in all positive results.

Blood and biochemical tests were performed including White blood cells (WBC), Lymphocytes, Red blood cells (RBC), Hemoglobin, Hematocrit, Creatinine (Cr), BUN, AST, ALT, Total bilirubin, Albumin, Platelet, LDH, PT(INR), Total cholesterol, HDL, Triglyceride (TG), Glucose, and CRP within the first 24 hours at admission. Elevated liver enzymes were considered clinically relevant if they reached >3 times the upper limit of normal.

### Procedures for treatment and measurement

Patients who had been tested positive for SARS-CoV-2 infection, were hospitalized within the first 24 hours. After admission, to reconfirm SARS-CoV-2 infection, nasal swab samples for RT-PCR tests were obtained from all patients. All patients received blood and biochemical tests within the first 24 hours at admission.

Those with radiologic bronchiolitis/pneumonia findings, chest x-rays (CXR) were taken on a regular basis until lesions were resolved. All CXR images were reviewed by experienced radiologists. The highest level of oxygen support each patient received during their hospitalization was recorded as well. Fever was recorded if a patient’s body temperature arose to 37.5°C or higher. Clinical information regarding all other COVID-19-related symptoms (feeling feverish, chill, cough, sputum, rhinorrhea, sore throat, myalgia, headache, diarrhea, dyspnea, and chest pain) or any adverse drug events were collected daily through telephone survey using pre-specified questionnaires. All baseline characteristics were measured at the time of admission into the hospital. If patients showed worsening of symptoms after the admission, blood and biochemical tests were proceeded again as follow-up.

Patients were further stratified by the severity of symptoms according to the National Institutes of Health (NIH) COVID-19 guideline. Individuals without shortness of breath, dyspnea, or abnormal imaging were categorized as mild COVID-19 (162 patients); individuals who had evidence of lower respiratory disease by clinical assessment, radiological imaging, or oxygen saturation (SaO_2_) >93% on room air at sea level were categorized as moderate COVID-19 (137 patients); individuals who had respiratory frequency >30 breaths per minute, SaO_2_ ≤93% on room air at sea level, ratio of arterial partial pressure of oxygen to fraction of inspired oxygen (PaO_2_/FiO_2_) <300, or lung infiltrates >50% were categorized as severe COVID-19 [39]. When mild and moderate patients progressed to the severe level, they were transferred to tertiary hospitals equipped with ICU rooms.

Considering the severity of COVID-19, antibiotics (Cefixime and Azithromycin) and antiviral agents (LoP/R or HQ) were administered when patients were suspected to have pneumonitis or bronchiolitis on CXR or showed symptoms related to lower respiratory tract such as dyspnea, shortness of breath, fever. Duration of treatment was 5–10 days depending on disease severity and clinical progression. Cefixime was prescribed until remission of pneumonia for 100mg tablets twice a day. Patients who were given HQ received 200mg HQ tablets twice a day. Patients who were given lopinavir/ritonavir received 200/50mg tablets twice a day. Azithromycin, when prescribed, was used for up to 5 days and given in the form of 500mg tablets once a day; most patients received azithromycin for 3 days. Standard supportive treatment included occasional injection of crystalloid solution, analgesics, non-steroidal antiinflammatory drugs.

Few patients initiated their treatment with LoP/R or hydroxychloroquine, but their treatment method was shortly switched to another one due to side effects such as nausea or progression of pneumonia; Among these patients, patients who received concurrent Lop/R and hydroxychloroquine more than 5 days, were excluded from the study.

48 hours after all the clinical symptoms were resolved, patients were tested again to check viral clearance of SARS-CoV-2. Complete viral clearance was affirmed by two consecutive negatives on RT-PCR at intervals of 24 hours. Negative conversion of viral load was defined when the cycle threshold (Ct) value exceeded 40 [38, 40]. After the confirmation of complete viral clearance, hospitalized patients were discharged. This decision was made according to the guidelines from Korea Centers for Disease Control and Prevention [41].

### Study outcomes

Our target patient population was hospitalized COVID-19 patients. Our primary endpoint was time to symptom resolution (i.e. time from the earliest date to the last date of any symptoms spotted) and our secondary endpoints were time to viral clearance (i.e. time from confirmation of SARs-CoV-2 infection to two consecutive negative results on PCR, signified by Ct value ≥ 40), hospital stay duration (i.e. time from admission to discharge from the hospitalization) and adverse effects (abnormal symptoms and abnormal laboratory indices).

### Statistical analysis

Continuous variables were reported as mean (standard deviation [SD]), and categorical variables were reported as number (percentage [%]). Continuous variables were analyzed using Student’s t-test or Mann-Whitney U test, and categorical data were compared using the chi-square test or Fisher’s exact test. P-values were reported from means for continuous variables and from percentages for categorical variables. Kaplan-Meier curves were generated for the primary and the secondary endpoints, and those were analyzed with the log-rank test. Cox proportional hazard ratio (HR) models were used to determine HRs and 95% confidence intervals (CIs). All tests were 2-sided, and P-values less than 0.05 were considered statistically significant. All analyses were conducted using pandas, sklearn and lifelines libraries in Python 3 and survminer and survival packages in R software, version 4.1.0. Multiple imputation was used to handle missing data, and model estimates and standard errors were calculated with Rubin’s rules [42].

#### Propensity score matching and Cox proportional hazards regression models

Propensity score matching was performed to balance the baseline characteristics of groups of patients in CA/LoP group, CA/HQ group and standard supportive group. Three rounds of propensity score matching was carried out to closely balance the baseline characteristics among the three groups **(Fig 1)**. Propensity scores were calculated using logistic regression with the following variables: sex, age, BMI, diagnoses of past history, current use of other medications, level of severity, systolic BP, Diastolic BP, heart rate, white blood cells, initial lymphocyte count, platelet, LDH, and CRP at admission. After computing the scores, matching was performed using the nearest-neighbor method. Patients in the two groups were matched at a 1:1 ratio based on their closest propensity score. When multiple patients in one group are duplicatively matched with one patient in another group, the patient pair with the closest propensity score out of multiple pairs is selected and the other patients are matched for the next closest matching patient. Matching is iteratively processed until all pairs with the propensity score difference within 0.5 are found, and remaining patients that could not find their conjugates are ignored **(S1 Fig)**. Cox proportional-hazards regression models were used to evaluate the association of the drug use with time to symptom resolution, time to viral clearance and hospital stay duration. Multivariable Cox regression model was performed with the same covariates used for the propensity-matching analysis.

## 3. Results

### Baseline demographics and initial laboratory indices of patients

A total of 299 patients were included in this study and the enrollment of the study cohort is described in **Fig 1**. Among 299 patients, 82 patients were administered with antibiotics (azithromycin and cefixime) and antiviral agents (LoP/R or HQ). Among 82 patients, 57 (19.0%) patients included in CA/LoP group, who received cefixime, azithromycin with lopinavir/ritonavir, 25 (8.3%) patients, included in CA/HQ group, who received cefixime, azithromycin with hydroxychloroquine. 217 patients (72.5%) received only the standard supportive care (Standard).

Baseline characteristics of the patients are displayed in **Table 1**. 130 (59.9%) were female in standard supportive therapy group, while 36 (63.16%) and 22 (88.0%) were female in CA/LoP and CA/HQ respectively. Compared with standard supportive group, there were more female patients in CA/HQ **(Table 1, P< 0.05)**. Between CA/LoP and CA/HQ, CA/HQ had more female patients **(Table 1, P < 0.05)**.

**Table 1:** Baseline characteristics, symptoms, comorbidities, vital signs, and initial laboratory indices of study cohorts before propensity score matching.

Before matching	Standard	CA/LoP	CA/HQ	P-value^a^	P-value^b^	P-value^c^
**Number of patients**	217	57	25			
**Baseline characteristics**						
Sex, Female(%)	130.0 (59.91)	36.0 (63.16)	22.0 (88.0)	0.768	0.011	0.044
Age	35.24 (14.21)	50.23 (13.38)	41.44 (14.93)	<0.001	0.041	0.01
BMI	22.95 (3.23)	24.35 (3.38)	23.02 (3.07)	0.004	0.919	0.094
Fever > = 37.5(%)	26.0 (11.98)	16.0 (28.07)	4.0 (16.0)	0.005	0.797	0.372
Mild and Asymptomatic(%)	160.0 (73.73)	2.0 (3.51)	0.0 (0.0)	<0.001	<0.001	0.864
Moderate(severity)(%)	57.0 (26.27)	55.0 (96.49)	25.0 (100.0)	<0.001	<0.001	0.864
Abnormal Radiological Finding	9.0 (4.15)	54.0 (94.74)	25.0 (100.0)	<0.001	<0.001	0.596
O2 supply application(%)	0.0 (0.0)	8.0 (14.04)	0 (0.0)	<0.001	1	0.117
**Symptoms**						
No symptoms	39.0 (17.97)	3.0 (5.26)	0.0 (0.0)	0.03	0.043	0.596
Feeling Feverish(%)	28.0 (12.9)	23.0 (40.35)	8.0 (32.0)	<0.001	0.025	0.638
Chill(%)	36.0 (16.59)	19.0 (33.33)	9.0 (36.0)	0.009	0.037	0.985
Cough(%)	93.0 (42.86)	36.0 (63.16)	17.0 (68.0)	0.01	0.029	0.864
Sputum(%)	106.0 (48.85)	43.0 (75.44)	18.0 (72.0)	0.001	0.048	0.957
Rhinorrhea(%)	97.0 (44.7)	30.0 (52.63)	15.0 (60.0)	0.358	0.215	0.707
Sore throat(%)	84.0 (38.71)	21.0 (36.84)	9.0 (36.0)	0.916	0.963	0.86
Myalgia(%)	46.0 (21.2)	31.0 (54.39)	9.0 (36.0)	<0.001	0.156	0.196
Headache(%)	87.0 (40.09)	29.0 (50.88)	12.0 (48.0)	0.188	0.585	1
Diarrhea(%)	56.0 (25.81)	28.0 (49.12)	13.0 (52.0)	0.001	0.012	1
Dyspnea(%)	38.0 (17.51)	23.0 (40.35)	10.0 (40.0)	<0.001	0.016	0.83
Chest pain(%)	39.0 (17.97)	20.0 (35.09)	6.0 (24.0)	0.009	0.644	0.462
Symptom counts	3.27 (2.47)	5.32 (2.85)	5.04 (2.59)	<0.001	0.001	0.68
**Comorbidities**						
Any Past History	40.0 (18.43)	16.0 (28.07)	3.0 (12.0)	0.155	0.603	0.192
Hypertension(%)	16.0 (7.37)	11.0 (19.3)	2.0 (8.0)	0.015	0.772	0.336
Diabetes mellitus(%)	6.0 (2.76)	1.0 (1.75)	0.0 (0.0)	0.967	0.871	0.67
Dyslipidemia(%)	6.0 (2.76)	3.0 (5.26)	1.0 (4.0)	0.6	0.779	0.755
Thyroid(%)	6.0 (2.76)	0.0 (0.0)	2.0 (8.0)	0.447	0.426	0.166
Cardiovascular disease*(%)	2.0 (0.92)	1.0 (1.75)	0.0 (0.0)	0.859	0.494	0.67
History of COPD, Asthma, Tuberculosis	6.0 (2.76)	0.0 (0.0)	0.0 (0.0)	0.447	0.871	1
Chronic Kidney disease	0.0 (0.0)	0.0 (0.0)	0.0 (0.0)	1	1	1
Malignancy, Cancer	1.0 (0.46)	1.0 (1.75)	1.0 (4.0)	0.883	0.494	0.864
Current Use of other Medications#	22.0 (10.14)	11.0 (19.3)	3.0 (12.0)	0.096	0.954	0.624
**Vital Signs**						
Systolic BP(mmHg)	128.49 (13.89)	129.33 (16.76)	127.0 (16.09)	0.698	0.617	0.559
Diastolic BP(mmHg)	77.35 (10.45)	77.44 (13.76)	78.4 (12.54)	0.96	0.643	0.766
Heart rate(/min)	86.7 (11.93)	87.3 (11.85)	85.68 (11.64)	0.736	0.685	0.569
Respiratory rate(/min)	20.05 (0.7)	20.02 (0.55)	20.16 (0.55)	0.741	0.453	0.285
Body temperature(°C)	36.97 (0.4)	37.18 (0.61)	37.06 (0.36)	0.002	0.275	0.367
**Laboratory indices**						
WBC(×10^9^ /L)	6.02 (1.53)	5.62 (1.74)	5.26 (1.43)	0.086	0.018	0.364
LYM(×10^9^ /L)	2.03 (0.52)	1.72 (0.53)	1.75 (0.54)	<0.001	0.012	0.833
RBC(×10^12^ /L)	4.68 (0.51)	4.52 (0.48)	4.45 (0.5)	0.032	0.032	0.543
Hemoglobin(g/dL)	14.0 (1.69)	13.8 (1.28)	13.35 (1.57)	0.4	0.070	0.181
Hematocrit(%)	42.09 (4.42)	41.46 (3.5)	40.47 (4.15)	0.318	0.083	0.271
Cr(mg/dL)	0.79 (0.18)	0.82 (0.21)	0.71 (0.14)	0.383	0.030	0.026
BUN(mg/dL)	12.34 (3.26)	12.98 (4.0)	11.57 (3.55)	0.213	0.268	0.134
AST(U/L)	24.59 (20.32)	28.95 (17.04)	23.92 (10.11)	0.138	0.872	0.174
ALT(U/L)	25.54 (22.54)	28.42 (34.99)	24.76 (23.64)	0.451	0.870	0.635
Total bilirubin(mmol/L)	0.55 (0.29)	0.71 (0.37)	0.59 (0.56)	<0.001	0.602	0.227
Albumin(g/dL)	4.36 (0.29)	4.02 (0.3)	4.12 (0.31)	<0.001	0.000	0.193
Platelet(×10^9^ /L)	267.78 (62.52)	258.16 (87.85)	259.0 (64.67)	0.346	0.508	0.966
LDH(U/L)	219.41 (76.64)	269.68 (74.49)	251.92 (120.92)	<0.001	0.062	0.418
Total cholesterol(mg/dL)	162.49 (33.17)	157.16 (33.8)	160.32 (26.24)	0.283	0.753	0.679
HDL(mg/dL)	46.28 (12.35)	41.82 (9.7)	45.14 (10.16)	0.012	0.658	0.162
TG(mg/dL)	162.94 (73.57)	140.54 (57.44)	146.08 (52.5)	0.034	0.267	0.681
Glucose(mg/dL)	94.3 (44.78)	119.21 (57.55)	94.64 (36.01)	0.001	0.971	0.052
CRP(mg/dL)	0.16 (0.3)	1.4 (1.94)	0.56 (1.14)	<0.001	0.000	0.049

BMI: body mass index; HQ: hydroxychloroquine; WBC:White blood cells; LYM:Lymphocytes; RBC:Red blood cells; AST: Aspartate aminotransferase; ALT: alanine aminotransferase; LDH: lactate dehydrogenase; BUN: blood urea nitrogen; HDL: high-density lipoproteins; PT (INR): prothrombin time (international normalized ratio); CRP: c-reactive protein; TG: triacyl-glyceride; SD: standard deviation. *Cardiovascular disease: coronary artery disease, Heart Failure, Arrhythmia. P-value^a^, p-value^b^, and p-value^c^ respectively represent test results of Standard vs CA/LoP groups, Standard vs CA/HQ groups, and CA/LoP vs CA/HQ groups. Continuous characteristics are shown as means and standard deviations in brackets, while categorical binary characteristics are shown as counts and percentages in brackets. P-values of continuous variables are based on t-test and p-values of categorical variables are based on chi-square test.

Mean age of patients was 35.24 years (standard deviation (SD) 14.21), 50.23 (SD 13.38), and 41.44 (SD 14.93) in standard supportive therapy group, CA/LoP group, and CA/HQ group respectively. Treatment groups (CA/LoP and CA/HQ) had more older patients than standard supportive group **(Table 1, CA/LoP P < 0.01, CA/HQ P < 0.05, respectively)**. Between CA/LoP and CA/HQ, CA/LoP had more of the elder population **(Table 1, P < 0.05)**.

Mean BMI of patients was 22.95 (SD 3.23), 24.35 (SD 3.38), and 23.02(SD 3.07) in standard supportive therapy group, CA/LoP group, and CA/HQ group respectively. CA/LoP showed significantly higher mean BMI rate than that in standard group **(Table 1, P < 0.01)**. However, there was no significant difference between CA/HQ and standard group **(Table 1)**. Also, There were no significant differences in any past history and current use of other medications among patients between treatment groups (CA/LoP and CA/HQ) and standard supportive group **(Table 1)**.

In terms of past history, there were more patients diagnosed with hypertension in CA/LoP compared with standard group. Except for hypertension, there were no differences between treatment groups and standard group in comorbidities at the baseline **(P < 0.05, Table 1)**. After propensity score matching, these baseline characteristics were not significantly different between the supportive therapy group and treatment groups (CA/LoP and CA/HQ) **(Table 2)**. Similarly, CA/LoP and CA/HQ groups showed no significant difference **(S1 Table)**.

**Table 2:** Baseline characteristics, symptoms, comorbidities, vital signs, and initial laboratory indices of Standard and CA/LOP group, CA/HQ group after propensity score matching.

After matching	Standard	CA/LoP	P-value	Standard	CA/HQ	P-value
**Number of patients**	25	25		15	15	
**Baseline characteristics**						
Sex, Female(%)	16.0 (64.0)	19.0 (76.0)	0.537	13.0 (86.67)	13.0 (86.67)	1
Age	46.84 (15.12)	45.08 (14.88)	0.68	45.2 (16.16)	41.07 (16.18)	0.49
BMI	23.49 (3.4)	23.8 (3.68)	0.759	22.92 (3.89)	23.28 (2.75)	0.767
Fever > = 37.5(%)	9.0 (36.0)	2.0 (8.0)	0.041	7.0 (46.67)	1.0 (6.67)	0.039
Mild and Asymptomatic(%)	6.0 (24.0)	2.0 (8.0)	0.247	1.0 (6.67)	0.0 (0.0)	1
Moderate(severity)(%)	19.0 (76.0)	23.0 (92.0)	0.247	14.0 (93.33)	15.0 (100.0)	1
Abnormal Radiological Finding	9.0 (36.0)	23.0 (92.0)	<0.001	7.0 (46.67)	15.0 (100.0)	0.004
O2 supply application(%)	0.0 (0.0)	0.0 (0.0)	1.0	0.0 (0.0)	0.0 (0.0)	1
**Symptoms**						
No symptoms	3.0 (12.0)	3.0 (12.0)	1.0	1.0 (6.67)	0.0 (0.0)	1
Feeling Feverish(%)	7.0 (28.0)	5.0 (20.0)	0.741	4.0 (26.67)	3.0 (20.0)	1
Chill(%)	4.0 (16.0)	3.0 (12.0)	1.0	2.0 (13.33)	5.0 (33.33)	0.388
Cough(%)	11.0 (44.0)	13.0 (52.0)	0.777	8.0 (53.33)	10.0 (66.67)	0.709
Sputum(%)	17.0 (68.0)	15.0 (60.0)	0.768	11.0 (73.33)	9.0 (60.0)	0.699
Rhinorrhea(%)	13.0 (52.0)	11.0 (44.0)	0.777	7.0 (46.67)	8.0 (53.33)	1
Sore throat(%)	14.0 (56.0)	8.0 (32.0)	0.154	8.0 (53.33)	4.0 (26.67)	0.264
Myalgia(%)	9.0 (36.0)	7.0 (28.0)	0.762	6.0 (40.0)	5.0 (33.33)	1
Headache(%)	15.0 (60.0)	11.0 (44.0)	0.396	8.0 (53.33)	7.0 (46.67)	1
Diarrhea(%)	9.0 (36.0)	12.0 (48.0)	0.567	4.0 (26.67)	8.0 (53.33)	0.264
Dyspnea(%)	11.0 (44.0)	4.0 (16.0)	0.064	8.0 (53.33)	3.0 (20.0)	0.13
Chest pain(%)	8.0 (32.0)	6.0 (24.0)	0.753	6.0 (40.0)	3.0 (20.0)	0.426
Symptom counts	4.72 (2.78)	3.8 (2.5)	0.224	4.8 (2.57)	4.33 (2.55)	0.622
**Comorbidities**						
Any Past History	6.0 (24.0)	4.0 (16.0)	0.724	4.0 (26.67)	2.0 (13.33)	0.648
Hypertension(%)	4.0 (16.0)	3.0 (12.0)	1.0	3.0 (20.0)	1.0 (6.67)	0.591
Diabetes mellitus(%)	1.0 (4.0)	0.0 (0.0)	1.0	0.0 (0.0)	0.0 (0.0)	1
Dyslipidemia(%)	0.0 (0.0)	0.0 (0.0)	1.0	0.0 (0.0)	1.0 (6.67)	1
Thyroid(%)	0.0 (0.0)	0.0 (0.0)	1.0	0.0 (0.0)	1.0 (6.67)	1
Cardiovascular disease*(%)	0.0 (0.0)	1.0 (4.0)	1.0	0.0 (0.0)	0.0 (0.0)	1
History of COPD, Asthma, Tuberculosis	1.0 (4.0)	0.0 (0.0)	1.0	1.0 (6.67)	0.0 (0.0)	1
Chronic Kidney disease	0.0 (0.0)	0.0 (0.0)	1.0	0.0 (0.0)	0.0 (0.0)	1
Malignancy, Cancer	0.0 (0.0)	0.0 (0.0)	1.0	0.0 (0.0)	1.0 (6.67)	1
Current Use of other Medications#	5.0 (20.0)	3.0 (12.0)	0.7	3.0 (20.0)	2.0 (13.33)	1
**Vital Signs**						
Systolic BP(mmHg)	128.28 (12.25)	129.72 (19.66)	0.757	125.67 (14.04)	128.53 (16.23)	0.609
Diastolic BP(mmHg)	77.96 (10.65)	77.68 (15.61)	0.941	77.2 (13.77)	78.8 (13.9)	0.754
Heart rate(/min)	84.88 (10.43)	83.48 (10.54)	0.639	83.27 (11.32)	85.53 (12.97)	0.614
Respiratory rate(/min)	19.92 (0.4)	19.84 (0.55)	0.561	19.87 (0.52)	20.13 (0.52)	0.168
Body temperature(°C)	37.1 (0.48)	36.9 (0.38)	0.123	37.15 (0.52)	36.95 (0.31)	0.199
**Laboratory indices**						
WBC(×10^9^ /L)	5.98 (1.43)	5.78 (1.73)	0.666	5.73 (1.26)	5.49 (1.51)	0.64
LYM(×10^9^ /L)	1.86 (0.48)	1.9 (0.44)	0.748	1.86 (0.53)	1.89 (0.57)	0.887
RBC(×10^12^ /L)	4.5 (0.53)	4.5 (0.42)	0.995	4.43 (0.41)	4.42 (0.52)	0.966
Hemoglobin(g/dL)	13.61 (1.85)	13.78 (1.07)	0.682	13.12 (1.52)	13.44 (1.73)	0.595
Hematocrit(%)	40.88 (5.0)	41.62 (2.85)	0.525	39.79 (4.03)	40.42 (4.66)	0.693
Cr(mg/dL)	0.78 (0.21)	0.79 (0.16)	0.845	0.69 (0.11)	0.71 (0.15)	0.779
BUN(mg/dL)	13.74 (4.26)	11.81 (2.88)	0.067	13.19 (4.0)	11.7 (3.58)	0.291
AST(U/L)	24.6 (12.19)	25.12 (19.19)	0.909	24.6 (13.66)	23.6 (10.51)	0.824
ALT(U/L)	26.48 (18.39)	29.88 (50.15)	0.752	24.8 (21.67)	26.93 (27.79)	0.816
Total bilirubin(mmol/L)	0.49 (0.3)	0.74 (0.42)	0.02	0.42 (0.28)	0.45 (0.22)	0.732
Albumin(g/dL)	4.25 (0.37)	4.15 (0.26)	0.283	4.31 (0.32)	4.14 (0.24)	0.126
Platelet(×10^9^ /L)	257.2 (66.87)	267.24 (74.27)	0.618	256.93 (55.87)	262.0 (70.0)	0.828
LDH(U/L)	238.0 (96.0)	228.44 (49.25)	0.66	244.4 (99.38)	224.47 (62.45)	0.516
Total cholesterol(mg/dL)	158.04 (32.15)	162.72 (34.67)	0.623	161.47 (34.81)	160.8 (28.7)	0.955
HDL(mg/dL)	41.86 (12.44)	43.42 (6.55)	0.58	44.89 (12.98)	45.71 (10.62)	0.852
TG(mg/dL)	164.6 (68.75)	131.36 (55.84)	0.067	152.27 (58.49)	141.73 (58.53)	0.626
Glucose(mg/dL)	96.08 (57.2)	113.44 (58.21)	0.293	83.2 (28.86)	97.4 (38.72)	0.264
CRP(mg/dL)	0.42 (0.81)	0.29 (0.33)	0.482	0.19 (0.23)	0.21 (0.18)	0.789

BMI: body mass index; HQ: hydroxychloroquine; WBC:White blood cells; LYM:Lymphocytes; RBC:Red blood cells; AST: Aspartate aminotransferase; ALT: alanine aminotransferase; LDH: lactate dehydrogenase; BUN: blood urea nitrogen; HDL: high-density lipoproteins; PT (INR): prothrombin time (international normalized ratio); CRP: c-reactive protein; TG: triacyl-glyceride; SD: standard deviation. *Cardiovascular disease: coronary artery disease, Heart Failure, Arrhythmia. Continuous characteristics are shown as means and standard deviations in brackets, while categorical binary characteristics are shown as counts and percentages in brackets. P-values of continuous variables are based on t-test and p-values of categorical variables are based on chi-square test.

Among 299 patients, 162 were mild and asymptomatic, while 137 were moderate according to the National Institutes of Health (NIH) COVID-19 guideline (24). In standard supportive therapy group, the majority of patients were asymptomatic or mild compared to those in treatment groups (CA/LoP and CA/HQ) **(Table 1, p < 0.01)**. Compared to standard supportive therapy group, treatment groups had significantly more number of moderate patients **(Table 1, p < 0.01)**. Between CA/LoP and CA/HQ, there was no significant difference in severity of disease **(Table 1)**. In terms of abnormal radiological findings and COVID-19 related symptom counts, there were significant differences between treatment groups (CA/LoP and CA/HQ) and standard supportive care group **(Table 1, P < 0.01),** while there was no difference in these properties between CA/LoP and CA/HQ **(Table 1)**. After matching, no significant difference in the severity of patients was observed neither between treatment groups and supportive therapy group **(Table 2)** nor between CA/LoP and CA/HQ **(S1 Table)**. Before matching, demographic factors, clinical symptoms, severity of disease, and laboratory indices were significantly different between the standard supportive therapy group and treatment groups. But after matching, most co-variables did not show significant differences **(Tables 1 and 2, S1 Table)**.

### General clinical outcomes and adverse reactions

General clinical outcome results are as follows: before propensity score matching, there were significant differences between standard supportive group and CA/LoP in all endpoints: transfer to tertiary hospital, time to viral clearance, time to symptom resolution, and hospital stay duration (**Table 3, P < 0.01**). Also, hospital stay duration in standard supportive group was significantly shorter than those in CA/HQ **(Table 3, P < 0.05),** but other endpoints did not show significant difference between standard group and CA/HQ **(Table 3)**. Between CA/LoP and CA/HQ, no endpoints showed significant difference. Only viral clearance duration was shorter in CA/HQ than CA/LoP, but it was not significant as well **(Table 3)**. After matching, none of the endpoints were significantly different between standard group and treatment groups **(Table 3)**. Also, between CA/HQ and CA/LoP, there was no significant difference in all endpoints **(S2 Table)**.

**Table 3:** Clinical outcomes before and after propensity score matching.

**Before matching**	**Standard**	**CA/LoP**	**CA/HQ**	**P-value** ^**a**^	**P-value** ^**b**^	**P-value** ^**c**^
**Number of patients**	217	57	25			
**Clinical outcomes**						
Tertiary hospital transfer(%)	7.0 (3.23)	9.0 (15.79)	1.0 (4.0)	0.001	0.7	0.256
Viral clearance(days)	20.42 (9.49)	27.84 (11.57)	22.92 (8.34)	<0.001	0.209	0.059
Hospital stay(days)	17.08 (9.83)	25.51 (11.81)	21.68 (8.24)	<0.001	0.026	0.146
Symptom resolution(days)	11.37 (11.87)	20.72 (15.76)	15.92 (11.17)	<0.001	0.069	0.172
**After matching**	**Standard**	**CA/LoP**	**P-value**	**Standard**	**CA/HQ**	**P-value**
**Number of patients**	25	25		15	15	
**Clinical outcomes**						
Tertiary hospital transfer(%)	1.0 (4.0)	1.0 (4.0)	1	1.0 (6.67)	1.0 (6.67)	1
Viral clearance(days)	23.32 (9.46)	23.88 (6.5)	0.808	25.67 (9.55)	26.2 (10.86)	0.887
Hospital stay(days)	20.88 (9.85)	21.44 (6.12)	0.81	24.4 (9.6)	23.27 (11.57)	0.772
Symptom resolution(days)	15.64 (12.72)	11.28 (8.45)	0.16	16.87 (13.37)	19.4 (14.1)	0.618

*for ICU admission, Psychiatric problem, failure to negative conversion of viral load before the closure of the hospital. P-value^a^, p-value^b^, and p-value^c^ respectively represent test results of Standard vs CA/LoP groups, Standard vs CA/HQ groups, and CA/LoP vs CA/HQ groups. P-values of continuous variables are based on t-test and p-values of categorical variables are based on chi-square test.

Adverse reaction results are as follows: before matching, CA/LoP showed more nausea, vomiting, and diarrhea symptoms compared with the standard supportive group **(Table 4, P < 0.01).** Patients in CA/HQ showed more diarrhea than those in the standard supportive group **(Table 4, P < 0.05)**, but such difference was not statistically significant. After matching, CA/LoP showed more nausea and vomiting than those of standard supportive group, but it was not significant as well **(S3 Table).** After matching, patients in CA/HQ showed significantly more diarrhea than those in the standard group **(S3 Table, P < 0.05).**

**Table 4:** Adverse effects and drug switch percentage before propensity score matching.

Before matching	Standard	CA/LoP	CA/HQ	P-value^a^	P-value^b^	P-value^c^
**Number of patients**	217	57	25			
**Adverse reactions**						
Nausea and Vomiting(%)	0.0 (0.0)	10.0 (17.54)	1.0 (4.0)	<0.001	0.192	0.192
Diarrhea(%)	56.0(25.81)	22.0(38.6)	8.0 (32.0)	0.082	0.671	1
Cardiac diseases*(%)	0.0 (0.0)	1.0 (1.75)	1.0 (4.0)	0.471	0.192	0.864
Psychological symptoms	1.0 (0.46)	1.0 (1.75)	0.0 (0.0)	0.883	0.192	0.67
Increased AST(%)	23.0 (10.6)	12.0(21.05)	5.0 (20.0)	0.06	0.289	0.851
Increased ALT(%)	43.0(19.82)	12.0(21.05)	6.0 (24.0)	0.983	0.818	0.994
Increased Total Bilirubin(%)	3.0 (1.38)	4.0 (7.02)	1.0 (4.0)	0.054	0.886	0.98
Increased Cr(%)	6.0 (2.76)	2.0 (3.51)	0.0 (0.0)	0.885	0.871	0.864
Increased BUN(%)	4.0 (1.84)	3.0 (5.26)	0.0 (0.0)	0.325	0.886	0.596
Increased LDH(%)	126.0 (58.06)	50.0(87.72)	17.0 (68.0)	<0.001	0.458	0.069
Increased CRP(%)	6.0 (2.76)	30.0(52.63)	7.0 (28.0)	<0.001	<0.001	0.068
**Drug switch**						
Switch from LoP/R to HQ(%)	0.0 (0.0)	0.0 (0.0)	2.0 (8.0)	1	0.003	0.166
Switch from HQ to LoP/R(%)	0.0 (0.0)	4.0 (7.02)	0.0 (0.0)	0.001	1	0.423
**Duration of medication use**						
Cefixime use(days)	0.0 (0.0)	9.3 (3.44)	8.45 (3.14)	<0.001	<0.001	0.279
AZ use(days)	0.0 (0.0)	3.69 (1.36)	2.8 (0.92)	<0.001	<0.001	0.003
LoP/R use(days)	0.0 (0.0)	8.48 (2.67)	0.24 (0.72)	<0.001	<0.001	<0.001
HQ use(days)	0.0 (0.0)	0.26 (0.97)	8.43 (2.29)	<0.001	<0.001	<0.001

*1 patient for cardiomegaly in CA/LOP group, 1 patient for tachycardia in CA/HQ group. P-value^a^, p-value^b^, and p-value^c^ respectively represent test results of Standard vs CA/LoP groups, Standard vs CA/HQ groups, and CA/LoP vs CA/HQ groups. P-values of continuous variables are based on t-test and p-values of categorical variables are based on chi-square test.

Regarding cardiac diseases, one patient was reported for cardiomegaly in CA/LoP, and one other patient was reported for tachycardia in CA/HQ. However, the incidence of cardiac diseases was not significantly associated with treatment group compared to standard group.

In laboratory indices, before matching, CA/LoP was significantly associated with increased levels of LDH and CRP compared with the standard supportive group **(Table 4, P < 0.01).** Also, CA/HQ was significantly associated with an increased level of CRP compared with standard supportive group **(Table 4, P < 0.01).** After matching, there was no significant difference in abnormally increased laboratory indices between treatment group and standard group **(S3 Table).**

Between CA/LoP and CA/HQ group, before matching, CA/LoP showed higher levels of CRP and LDH compared to those of CA/HQ but it was statistically insignificant **(Table 4).** After matching, the incidence of abnormal laboratory indices between two groups was not significantly different **(S3 Table).** Both before and after matching, CA/LoP received significantly longer azithromycin treatment than CA/HQ in terms of duration in medication usage (**Table 4, S4 Table, P < 0.01**).

### Treatment response

**Fig 2:**
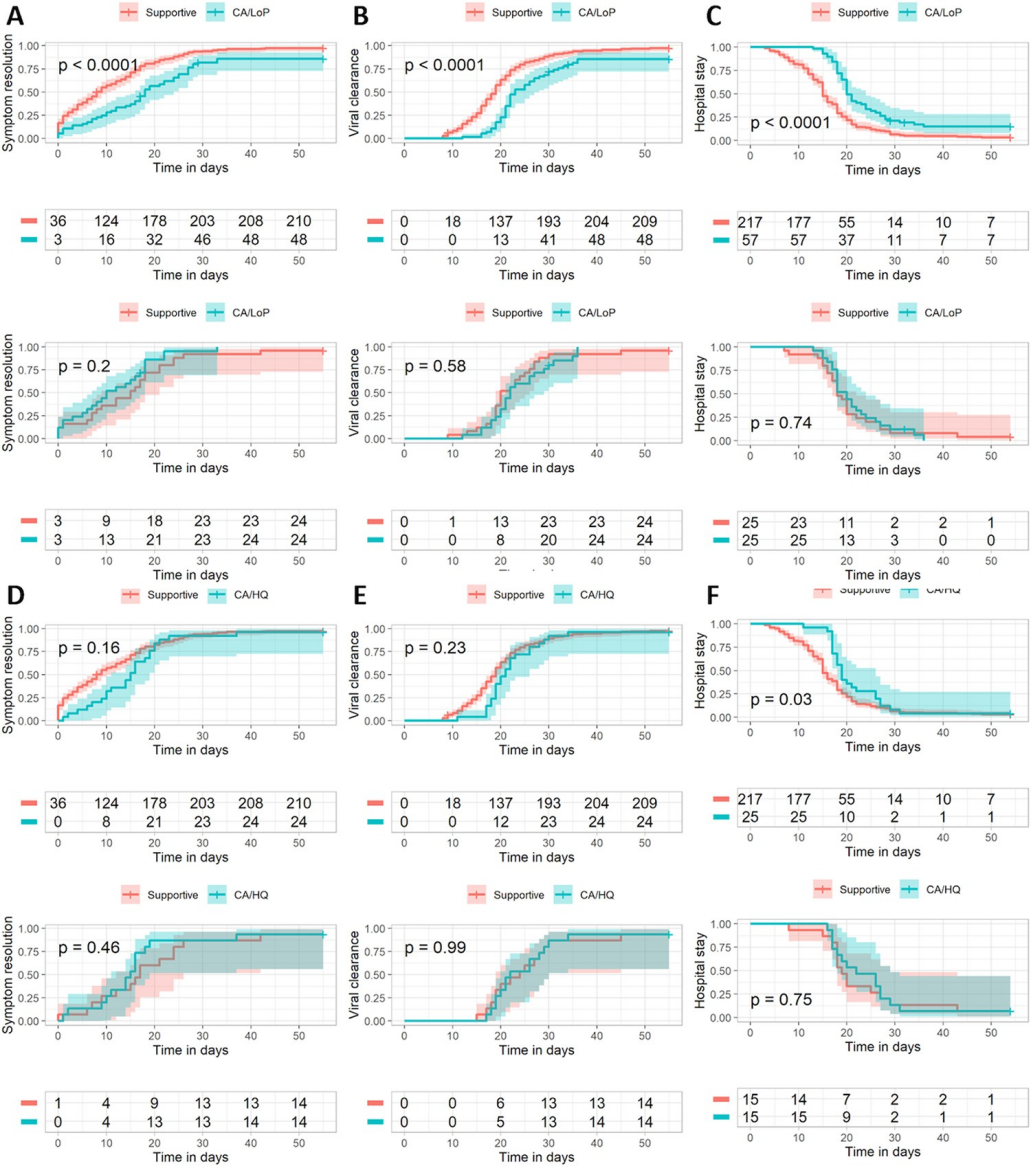
Kaplan-Meier curves regarding the three endpoints: Time to symptom resolution, time to viral clearance, and hospital stay duration, comparing CA/LoP vs standard group (A-C) and CA/HQ vs standard group (D-F). A-C: CA/LoP vs Standard group D-F: CA/HQ vs Standard group A, D: Time to Symptom resolution B, E: Time to viral clearance C, F: Hospital stay duration.

In overall cohort (n = 299), before matching, CA/LoP showed significantly longer time to symptom resolution (**Table 5, HR = 0.499(95% CI = 0.365–0.684))**, time to viral clearance (**Table 5, HR = 0.491(95% CI = 0.359–0.671)),** and hospital stay duration (**Table 5, HR = 0.455(95% CI = 0.333–0.623))** compared to those of standard group in both crude Cox regression analysis and Kaplan-Meier curves **(log-rank test, p < 0.01, Fig 2A–2C, respectively)**. However, after propensity score matching, these significant differences were not observed in Kaplan-Meier curves (**Fig 2A–2C)**. Also, in multivariable cox regression analysis, cox regression analysis after matching, and cox regression analysis adjusted for calculated propensity score, no significant differences were observed between the two groups **(Table 5)**.

**Table 5:** Associations of antibiotics and antiviral treatments with time to symptom resolution, time to viral clearance, and hospital stay duration in crude analysis, multivariable analysis, and propensity score matching analysis. Standard therapy group is used as the reference.

	Standard vs CA/LoP		Standard vs CA/HQ	
	Hazard Ratio (95% CI)	P-value	Hazard Ratio (95% CI)	P-value
**Time to symptom resolution**				
Crude, unadjusted cox regression	0.499 (0.365–0.684)	<0.001	0.741(0.486–1.127)	0.162
Multivariable Cox regression*	0.894 (0.559–1.431)	0.641	1.092(0.611–1.953)	0.766
Propensity-score analyses				
With matching**	1.447 (0.813–2.577)	0.209	1.331(0.631–2.809)	0.453
Adjusted for propensity score***	1.014 (0.604–1.701)	0.958	1.405(0.693–2.857)	0.346
**Time to viral clearance**				
Crude, unadjusted cox regression	0.491(0.359–0.671)	<0.001	0.786 (0.516–1.196)	0.261
Multivariable Cox regression*	0.610(0.387–0.962)	0.033	1.076 (0.612–1.890)	0.800
Propensity-score analyses				
With matching**	0.861(0.485–1.527)	0.608	1.005 (0.480–2.105)	0.990
Adjusted for propensity score***	0.646(0.394–1.060)	0.084	1.011 (0.501–2.037)	0.976
**Hospital stay duration**				
Crude, unadjusted cox regression	0.455(0.333–0.623)	<0.001	0.636 (0.418–0.969)	0.035
Multivariable Cox regression*	0.665(0.422–1.047)	0.078	1.045 (0.596–1.832)	0.879
Propensity-score analyses				
With matching**	0.902(0.510–1.595)	0.723	0.887 (0.422–1.862)	0.750
Adjusted for propensity score***	0.733(0.447–1.200)	0.217	0.919 (0.457–1.848)	0.813

* Hazard ratio from the multivariable Cox proportional hazards model where sex, age, BMI, diagnoses of past history, current use of other medications, level of severity, systolic BP, Diastolic BP, heart rate, white blood cells, initial lymphocyte count, platelet, LDH, and CRP at admission were the covariates

** Hazard ratio from a multivariable Cox proportional hazards model with the same covariates on the matched data set

*** Hazard ratio from a multivariable Cox proportional hazards model with the propensity score as an additional covariate.

Between CA/HQ group and standard group, before matching, CA/HQ showed significantly longer hospital stay duration in both unadjusted cox regression analysis **(HR 0.636 (95% CI = 0.418–0.969), Table 5)** and Kaplen-Meier curves **(log-rank test, p < 0.05, Fig 2F),** but all other endpoints did not show significance even before matching between two groups **(Fig 2D and 2E)**. After matching, every endpoint was not significantly associated with CA/HQ **(Fig 2D–2F)**. Similar results were observed in multivariable cox regression analysis, cox regression analysis adjusted for calculated propensity score, and cox regression analysis after matching **(Table 5)**.

Between CA/LoP and CA/HQ, CA/HQ showed shorter time to symptom resolution and viral clearance in Kaplan-Meier curves before matching. **(P < 0.05, S2 Fig)**. In unadjusted cox regression analysis, CA/HQ was more likely to achieve shorter time to viral clearance **(HR = 1.788 (95% CI = 1.092–2.924), S5 Table)**. However, after matching, the significant difference between two groups was no longer observed **(S2 Fig)**. Similar results were observed in multivariable cox regression analysis, cox regression analysis adjusted for calculated propensity score, and cox regression analysis after matching **(S5 Table)**.

## 4. Discussion

In this study cohort, most patients were classified as non-severe similarly to those in other cohorts of North-East Asian countries **(Table 1)** [30, 43]. According to the quarantine standards in South Korea, at the very start of the outbreak, large-scale mass-screening was carried out to detect patients to promptly detect and respond to this novel yet serious pandemic situation [31]. Even if a person did not show any symptom related to COVID-19 infection, the person was procedurally required to take RT-PCR screening if the person was in an enclosed space with any other person who is COVID-19 positive. Patients confirmed positive in COVID-19 RT-PCR test were kept in quarantine until RT-PCR results showed negative twice within 24 hours in a row. The guideline set by KDCA (Korean Disease Control and Prevention Agency) required to quarantine any individuals confirmed positive from RT-PCR without considering the severity or the intensity of symptoms, so mild-to-moderate patients who would not voluntarily get tested were also quarantined [41]. In addition, Korea Worker’s Compensation & Welfare Service Daegu Hospital, from which data of this study was collected, was an emergency center to contingently respond to the sudden shortage of quarantine wards, so there was no ICU ward to treat patients with severe COVID-19. Patients who showed progressive aggravation on the severity of COVID-19 were transferred to a tertiary hospital equipped with the ICU ward **(Table 3)**. In regard to the quarantine standard of South Korea and the peculiarity of this center, population of the cohort group mainly consisted of mild to moderate COVID-19 category in terms of severity.

Although all patients included in this study were mild and moderate, there was a significant difference between treatment group and standard group in the severity of the disease. Patients in treatment group were more likely to be diagnosed moderate but those in the standard were most likely diagnosed as mild and asymptomatic **(P < 0.01, Table 1)**. In this study, combination of antibiotics (cefixime and azithromycin) and antiviral agents (either LoP/R or HQ) was administered to patients under the discretion of clinicians. Under EUA (Emergency Use Authorization), using the combination of antibiotics with antiviral agents to COVID-19 patients was allowed based on prior observational studies. However, this authorization was not a formal approval to use these drugs on the new disease because there was insufficient evidence in clinical trials at the time the cohort received these treatments. Accordingly, clinicians were careful when using these old repositioned drugs for new purposes considering benefit and risk. Thus, the administration of the drugs was determined by clinicians based on subjective and objective evidence such as the severity of disease, abnormal laboratory indices, radiological findings including pneumonitis and pneumonia, lower respiratory tract clinical symptoms including dyspnea and chest pain, and the possibility of disease progression. As a result, drugs were mostly prescribed to patients with these proxies of severity, and most of the patients included in the treatment group were more severe than those in the standard group **(P < 0.01, Table 1);** this explains the different baseline characteristics between treatment group and standard group. Patients included in CA/LoP group were significantly older, had higher BMI, showed more COVID-19 related symptoms **(P < 0.01, Table 1)**, and had more hypertension **(P < 0.05 Table 1)** than those in standard group. Also, in laboratory indices, baseline levels of WBC, Lymphocyte, Albumin, LDH, CRP, etcs were higher for patients in CA/LoP group than those in standard group **(Table 1)**. Similar results were also observed between CA/HQ group and the standard group **(Table 1)**. Patients in CA/HQ group had higher age, higher body mass index (BMI), more existence of current comorbidities, and increased levels of WBC, LDH and CRP, all of which were closely associated with critical illness in COVID-19 patients [44]. Specifically, higher BMI was associated with higher risk of COVID-19 infection, mechanical ventilation and severe pulmonary complications [45]. In addition, comorbidities such as hypertension, diabetes, osteoporosis, chronic lower respiratory, chronic renal failure, and end-stage renal disease were significantly associated with the severity of COVID-19 in South Korean population [46]. Thus, patients who were treated with antibiotics and antiviral agents were more likely to be older, have higher BMI, more likely to be diagnosed with comorbidities, and more likely to be observed with deteriorated levels of laboratory indices. In addition, CA/LoP and CA/HQ groups were associated with longer time to symptom resolution, time to viral clearance and hospital stay duration compared to those of standard group before propensity score matching analysis **(Fig 2, Table 5)**. These results can be explained from the difference in baseline characteristics of treatment and standard groups, that older and more susceptible patients were treated with antibiotics and antiviral agents, while standard group mostly consisted of patients with mild symptoms. After matching and controlling the baseline characteristics, the statistical significance in the endpoints no longer existed **(Fig 2, Table 5)**.

In this study, three endpoints—time to symptom resolution, time to viral clearance, and hospital stay duration—showed similar results between treatment group and standard group. **(Fig 2)** Three endpoints tend to be associated with the shorter results in the standard group than those in the treatment group before matching. However, after matching, these significant differences disappeared in all endpoints. Three endpoints were closely related as these are measured as follows. As mentioned in the method section, after 48 hours all clinical symptoms were resolved (time to symptom resolution), complete viral clearance was confirmed by two consecutive negatives on RT-PCR by nasopharyngeal swab at 24 hour intervals (time to viral clearance). After the confirmation of viral clearance, the patients were discharged (hospital stay duration). Relationship among the three endpoints was also observed in other studies as well. Low SARS-CoV-2 Ct values confirmed by RT-PCR via nasopharyngeal swab were correlated with the increased probability of progression to severe disease [47], increased disease severity [48], presence of abnormal radiological findings in chest imaging [49], and presence of biochemical and haematological markers [50] in COVID-19 patients. Thus, similarity of three endpoints between treatment group and standard group are supported by both prior literature and procedural methodology applied in this study.

Cephalosporin and azithromycin were the most commonly used antibacterial prophylactics and treatment against bacterial coinfections in SARS-CoV-2 patients, but there still lack literature on their exact indications and efficacy [36]. In addition, these antibiotics with antiviral agents were often used without solid microbiological evidence on bacterial coinfections followed by COVID-19 infections in mild to moderate patients [51]. In Townsend’s study, which assessed the rate of bacterial coinfections in COVID-19 patients by using microbiological sampling such as sputum culture, blood cultures and urinary antigen testing for *Streptococcus pneumoniae* and *Legionella pneumophila*, 117 patients received prolonged course of antimicrobial therapy and showed lower respiratory tract symptoms despite a low rate of bacterial coinfection in SARS-CoV-2. Only 6% of patients who were identified with respiratory pathogens needed to be treated with antimicrobial therapy [52]. Hugh Adler and Rober Ball also reported that only three percent of hospitalized SARS-CoV-2 patients had the evidence of pneumococcal coinfection, and only one of 31 patients was confirmed with positive for *Legionella* antigen [53]. Timothy M. Rawson highlighted that broad-spectrum empirical antibiotics were frequently prescribed to patients with coronavirus-associated respiratory infections which include SARS-CoV-2, SARS-1, MERS, and other coronaviruses, although there was not enough data to support its association with respiratory bacterial/fungal coinfection [54]. These studies emphasized the necessity of using microbiological sampling in SARS-CoV-2 patients and the better rationale on prescribing of antibiotics and appropriate stewardship interventions. In alignment with the previous studies, this study concludes that combination of cefixime and azithromycin with either LoP/R or HQ in moderate COVID-19 patients did not show statistical difference in time to symptom resolution, time to viral clearance, and therefore hospital stay duration compared to those of standard group after matching **(Fig 2)**. Thus, antibiotics should be administered with discretion to treat bacterial coinfection supported with microbial evidence, although pneumonia in radiological findings, symptoms of low respiratory tract infection, increased levels of WBC, LDH, CRP and etcs were observed in moderate SARS-CoV-2 patients. Likewise, several clinical trials reported that antiviral agents including LoP/R and hydroxychloroquine were not associated with significant clinical improvement and viral clearance in mild to moderate covid-19 patients [55] as our study also concluded.

There have been concerns on the safety of simultaneously using antiviral agents and antibiotics, and specifically the combination of hydroxychloroquine and azithromycin has raised safety concerns such as abnormal ECG, QTc prolongation, torsade pointes, and cardiac arrest [35, 56]. Also, in clinical trials, severe SARs-CoV-2 patients who received high-dose chloroquine (600mg twice daily for 10 days) showed prolongation of QTc interval compared to those who received low-dose chloroquine (450mg twice daily on day 1 and once daily for 4 days) in the study of Mayla, Fernando, Vanderson et al [57]. However, in our study, only one patient showed tachycardia in CA/HQ group **(Table 4)**, and after matching, there was no significant difference in heart-related problems between CA/HQ and standard group **(S3 Table).** Furthermore, no other serious adverse effects were observed in CA/HQ group. **(Table 4)** It is assumed that this discrepancy was attributed to three reasons. First, compared with Chloroquine (CQ) used in Mayla’s study, hydroxychloroquine (HQ) has been known for fewer side effects and safety in pregnancy [58]. Because of a lower level of tissue accumulation compared with CQ [59], the maximum tolerable dose for HQ is 1200mg, in contrast to chloroquine for which the maximum tolerable dose is 500mg [60]. Moreover, retinopathy and cardiomyopathy, the well-known side effects of these drugs, occurred less likely in HQ administration unless provided in high-dose and in took for long-term [59]. Second, in this study cohort, CA/HQ group received 200mg twice a day, which is lower than those from other HQ-related studies [35, 61]. Generally, severe side effects caused by HQ tend to be dose-dependent [57]. Third, patients in our cohort had much less severity of disease. QT prolongation may be influenced by patients attributes such as presence of comorbidities and the severity of disease [62]. Result of this study was consistent with Sarah M and Melanie’s study which reported no serious side effect using hydroxychloroquine as a postexposure prophylaxis and early treatment for asymptomatic or mild COVID-19 outpatients [63]. Our study did not find that the combination of cefixime, azithromycin and low-dose hydroxychloroquine was not associated with serious side effects in moderate COVID-19 patients.

In addition, patients in CA/LoP group showed more gastrointestinal symptoms (nausea, vomiting and diarrhea) compared with those in standard group before and after matching **(Table 4, S3 Table).** This side effect was also frequently reported in other clinical studies as well [34, 64]. Similar to other studies which investigated efficacy and safety of treating LoP/R in COVID-19 patients, this study also showed that none of patients in CA/LoP group showed serious adverse effects, but only few patients reported an increase in liver enzyme though with no statistical significance and elevated levels of CRP and LDH before matching. In sum, results of this study support that a combination of cefixime, azithromycin with either low dose HQ or LoP/R does not cause serious side effects in moderate COVID-19 patients who does not have many comorbidities which are related to the severe progression of COVID-19.

This study has several limitations. First, the characteristics of retrospective cohort study intrinsically presents selection bias in both internal and external validity. Unlike clinical trials, this study was conducted after the hospital data was recorded, so the observers could decide the cohort as intended. To mitigate this bias and to improve internal validity, we included patients hospitalized in a single-quarantine facility as much as possible other than 50 patients from other hospitals where different medical staffs worked and different ways of medical records were used, 9 patients who received different treatment such as only antibiotic administration or concurrent use of antiviral agents. Since this study cohort showed similar characteristics that have been reported from other studies, this study maintains good external validity [35, 65]. In addition, we tried not to remain an “unmeasured confounder” which can be a real problem when applying propensity-score matching (PSM) analysis and included the key confounding variable for treatment. Since clinicians decided antimicrobial and antiviral administrations based on the severity of disease, there was a confounding factor that had to be balanced before analyzing to avoid the selection bias. The severity of disease at admission was determined by the clinical symptoms and radiological findings, which implied lower respiratory disease such as pneumonia. These confounding variables are all included for PSM. As long as there are no unmeasured confounders, PSM would mitigate selection bias [66]. Other retrospective studies also showed baseline differences between treatment [67, 68] and standard group before PSM and ran statistical analysis on the data after matching where these differences are mostly mitigated [69]. Second, this study examined a relatively small number of patients. However, the cohorts in this study were closely observed for a long period of time from admission to discharge at the quarantine facility, which we believe is sufficiently significant in terms of total time observed compared with the other studies. Third, the cohorts in this study were heterogeneous. Different baseline characteristics among the cohorts might hinder objective evaluation of efficacy and safety of drug combinations. In order to balance the baseline characteristics among cohorts, this study applied various statistical analyses such as propensity-score matching, multivariate Cox regression analysis, and Cox regression analysis with propensity-score as a covariate. After controlling the effects of covariates among the cohorts, the difference in every endpoint between treatment group and standard group was not statistically significant. Fourth, since EKG (Electrocardiogram) was not closely observed in the cohorts in this study, it is difficult to assert that combination of azithromycin and hydroxychloroquine does not trigger coronary issues such as QT prolongation, the well-known serious side effect. Instead, hospitalized patients were closely observed through vital sign monitoring of heart rate and blood pressure and abnormal radiological findings related to heart from the x-ray analysis. As a result, only one case of cardiomegaly was spotted in the CA/LoP group, and only one tachycardia was spotted in the CA/HQ group. No statistical significance was found between treatment group and standard group, and neither did any serious adverse effects across all cohorts. Fifth, microbiological evidence has not been closely observed in this study. When data on the cohort of this study was collected, microbiological evidence on empirical antibiotics used for treating COVID-19 patients was not necessarily considered as important as it is nowadays. Although microbiologically evident tests such as blood culture, urine culture, urine antigen test, and bronchoalveolar lavage were not proceeded, groups in this study were uniquely designed by dividing the patients into antibiotic-administered treatment group and non-antibiotic standard supportive group. Whereas other COVID-19 studies did not divide cohorts into groups with or without antibiotics or considered third-generation antibiotics treatments as supportive [34, 65], this study meticulously observed the clinical prognosis by comparing endpoints between antibiotic and non-antibiotic groups for a sufficient period of time. We believe that lack of microbiological evidence was offset by sufficient clinical evidence.

## 5. Conclusion

In summary, a combined treatment of 3rd cephalosporin, azithromycin, and either low-dose lopinavir/ritonavir or hydroxychloroquine was not associated with better clinical outcomes and did not reduce time to viral clearance, time to symptom resolution, and hospital stay duration compared to those of conservative treatment in moderate COVID-19 patients. Thus, microbiological evidence should be closely monitored when using antibiotics in SARS-CoV-2 patients to prevent indiscreet administration of empirical antimicrobial treatments.

## Supporting information

S1 Fig Distribution of estimated propensity scores among cohorts. (TIF)

S2 Fig Kaplan-Meier curves regarding the three endpoints: Time to symptom resolution, time to viral clearance, and hospital stay duration, comparing CA/LoP and CA/HQ group. (TIF)

S3 Fig Kaplan-Meier curves regarding the tertiary transfers comparing A. control and CA/HQ group B. control and CA/LoP group C. CA/LoP and CA/HQ group.
(TIF)

S1 Table Baseline characteristics, symptoms, comorbidities, vital signs, and initial laboratory indices of CA/LoP and CA/HQ group after propensity score matching. (DOCX)

S2 Table Clinical outcomes of CA/LoP and CA/HQ groups after propensity score matching. (DOCX)

S3 Table Adverse effects and drug switch percentage after propensity score matching. (DOCX)

S4 Table Adverse effects and drug switch percentage of CA/LoP and CA/HQ groups after propensity score matching. (DOCX)

S5 Table Comparison between CA/HQ and CA/LoP groups in terms of time to symptom resolution, time to viral clearance, and hospital stay duration in crude analysis, multivariable analysis, and propensity score matching analysis. CA/LoP group is used as the reference.
(DOCX)
