# Supplementary figures and images for: Use of combined treatment of 3rd-generation cephalosporin, azithromycin and antiviral agents on moderate SARs-CoV-2 patients in South Korea: A retrospective cohort study
Source: PLoS One. 2022 May 4;17(5):e0267645. doi: 10.1371/journal.pone.0267645 (PMC9067652; doi:10.1371/journal.pone.0267645)

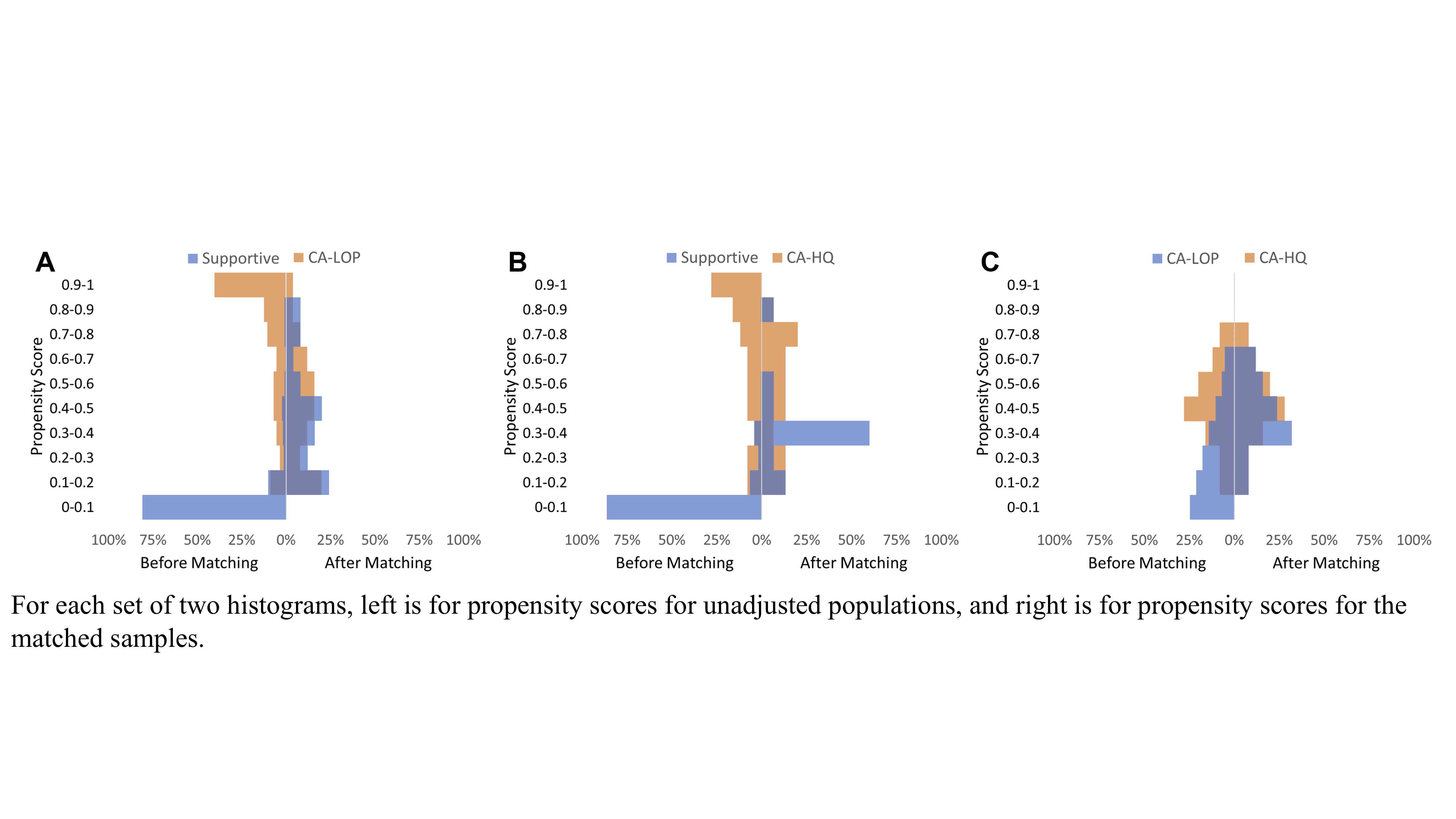

Supplement: S1 Fig — (TIF) [file pone.0267645.s001.tif]

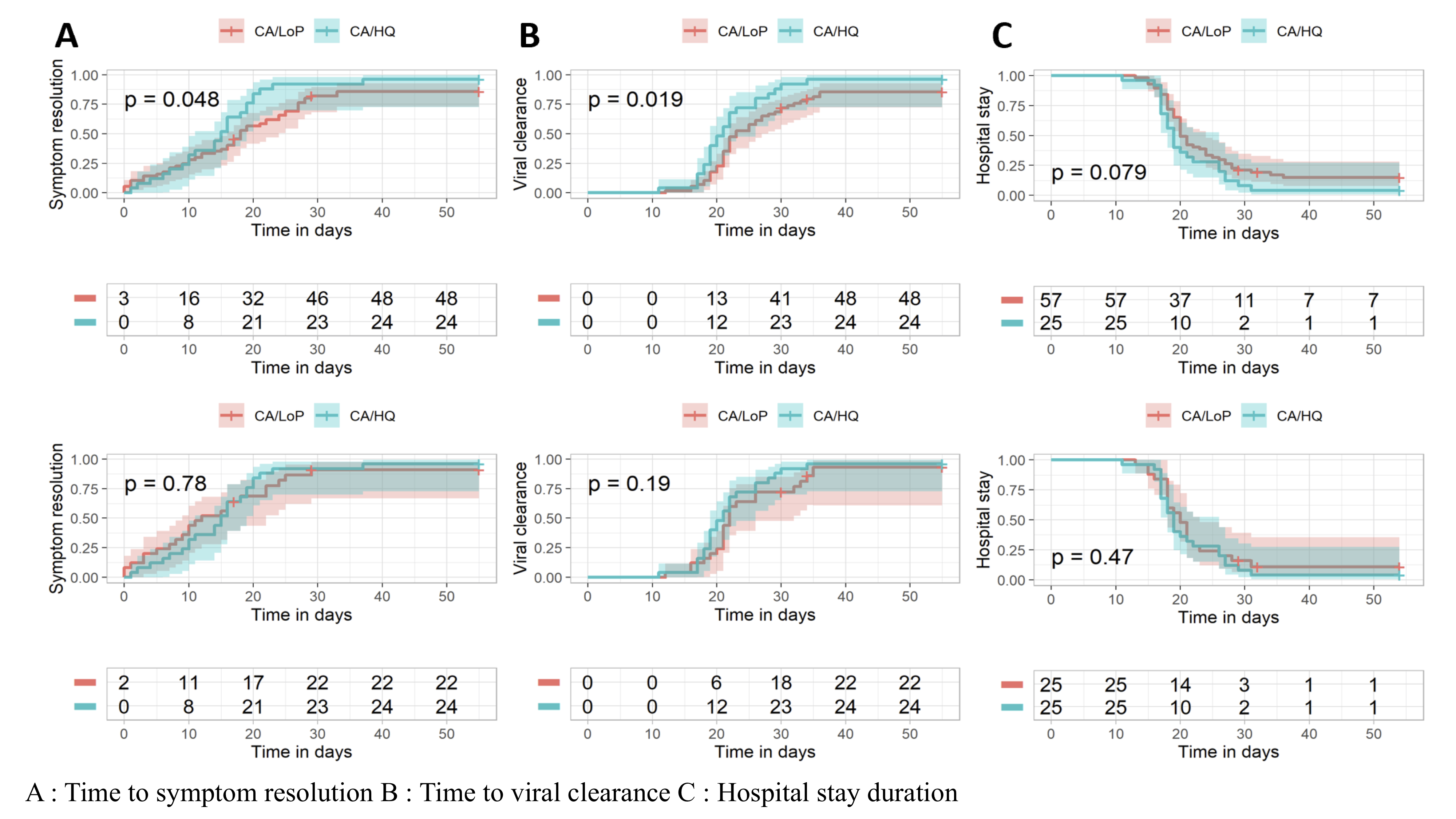

Supplement: S2 Fig — (TIF) [file pone.0267645.s002.tif]

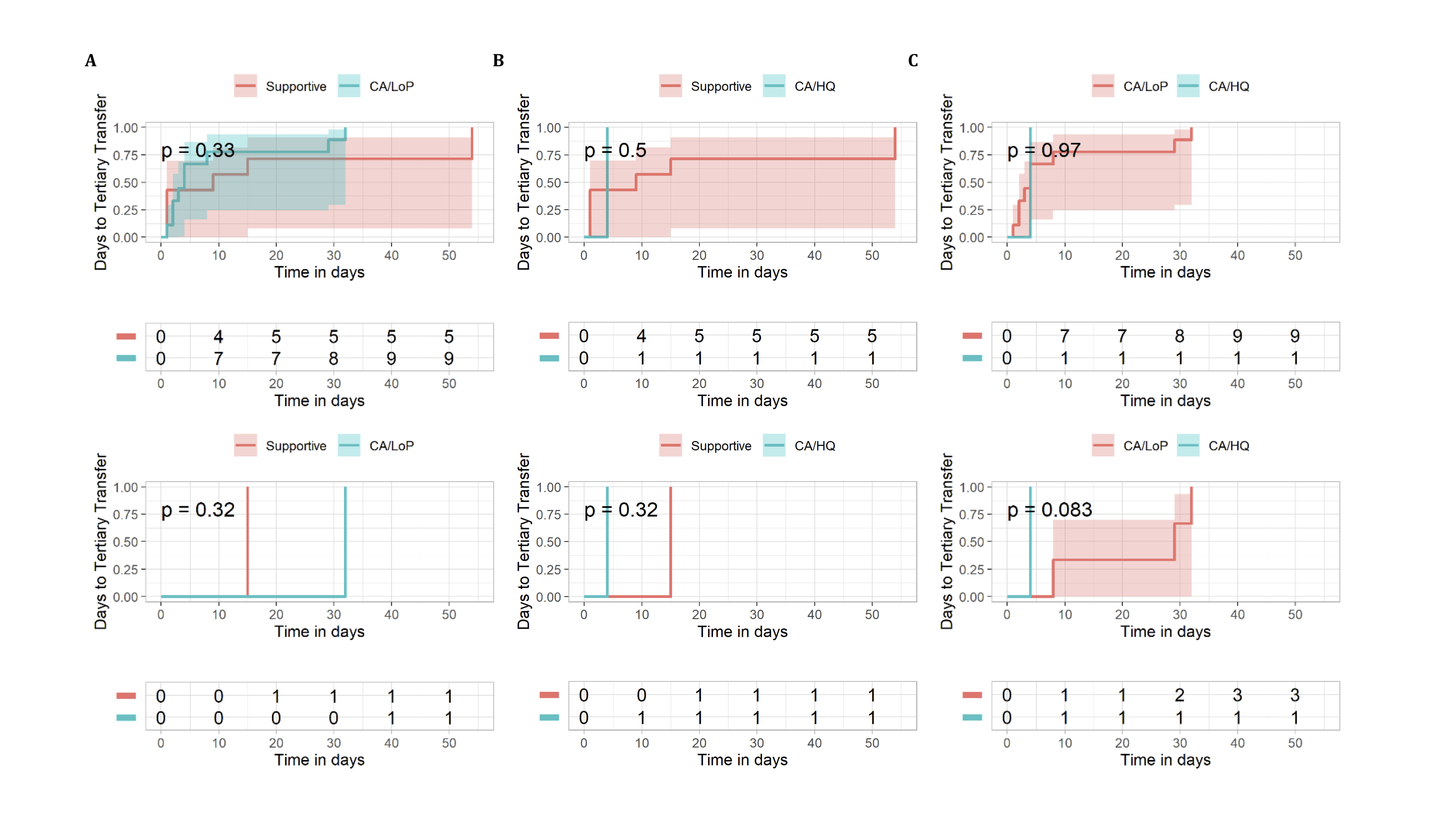

Supplement: S3 Fig — Kaplan-Meier curves regarding the tertiary transfers comparing A. control and CA/HQ group B. control and CA/LoP group C. CA/LoP and CA/HQ group. (TIF) [file pone.0267645.s003.tif]
