# Supplementary material for: Use of combined treatment of 3rd-generation cephalosporin, azithromycin and antiviral agents on moderate SARs-CoV-2 patients in South Korea: A retrospective cohort study
Source: PLoS One. 2022 May 4;17(5):e0267645. doi: 10.1371/journal.pone.0267645 (PMC9067652; doi:10.1371/journal.pone.0267645)
Supplement: S1 Table — (DOCX) [file pone.0267645.s004.docx]

**Supplementary table 1. Baseline characteristics, symptoms, comorbidities, vital signs, and initial laboratory indices of CA/LoP and CA/HQ group after propensity score matching.**

| **After matching** | **CA/LoP** | **CA/HQ** | **P-value** |  | **CA/LoP** | **CA/HQ** | **P-value** |
| --- | --- | --- | --- | --- | --- | --- | --- |
| **Number of patients** | 25 | 25 |  | Cardiovascular disease*(%) | 0.0 (0.0) | 0.0 (0.0) | 1 |
| **Baseline characteristics** |  |  |  | History of COPD, Asthma, Tuberculosis(%) | 0.0 (0.0) | 0.0 (0.0) | 1 |
| Sex, Female(%) | 23.0 (92.0) | 22.0 (88.0) | 1 | Chronic Kidney disease(%) | 0.0 (0.0) | 0.0 (0.0) | 1 |
| Age | 45.0 (14.64) | 41.44 (14.93) | 0.399 | Malignancy, Cancer(%) | 0.0 (0.0) | 1.0 (4.0) | 1 |
| BMI | 23.15 (2.81) | 23.02 (3.07) | 0.879 | Current Use of other. Medications#(%) | 4.0 (16.0) | 3.0 (12.0) | 1 |
| Fever >= 37.5(%) | 6.0 (24.0) | 4.0 (16.0) | 0.724 | **Vital Signs** |  |  |  |
| Mild and Asymptomatic(%) | 0.0 (0.0) | 0.0 (0.0) | 1 | Systolic BP(mmHg) | 127.32 (19.32) | 127.0 (16.09) | 0.95 |
| Moderate (severity)(%) | 25.0 (100.0) | 25.0 (100.0) | 1 | Diastolic BP(mmHg) | 77.12 (16.02) | 78.4 (12.54) | 0.754 |
| Abnormal Radiological Finding | 25.0 (100.0) | 25.0 (100.0) | 1 | Heart rate(/min) | 86.56 (10.66) | 85.68 (11.64) | 0.782 |
| **Symptoms** |  |  |  | Respiratory rate(/min) | 20.0 (0.0) | 20.16 (0.55) | 0.155 |
| No symptoms | 2.0 (8.0) | 0.0 (0.0) | 0.47 | Body temperature(℃) | 37.14 (0.58) | 37.06 (0.36) | 0.601 |
| Feeling Feverish(%) | 8.0 (32.0) | 8.0 (32.0) | 1 | **Laboratory indices** |  |  |  |
| Chill(%) | 6.0 (24.0) | 9.0 (36.0) | 0.537 | WBC(×10^9^ /L) | 5.26 (1.37) | 5.26 (1.43) | 0.998 |
| Cough(%) | 15.0 (60.0) | 17.0 (68.0) | 0.768 | LYM(×10^9^ /L) | 1.71 (0.37) | 1.75 (0.54) | 0.769 |
| Sputum(%) | 18.0 (72.0) | 18.0 (72.0) | 1 | RBC(×10^12^ /L) | 4.44 (0.46) | 4.45 (0.5) | 0.923 |
| Rhinorrhea(%) | 12.0 (48.0) | 15.0 (60.0) | 0.57 | Hemoglobin(g/dL) | 13.5 (1.17) | 13.35 (1.57) | 0.715 |
| Sore throat(%) | 9.0 (36.0) | 9.0 (36.0) | 1 | Hematocrit(%) | 41.02 (3.36) | 40.47 (4.15) | 0.612 |
| Myalgia(%) | 11.0 (44.0) | 9.0 (36.0) | 0.773 | Cr(mg/dL) | 0.76 (0.14) | 0.71 (0.14) | 0.24 |
| Headache(%) | 12.0 (48.0) | 12.0 (48.0) | 1 | BUN(mg/dL) | 11.36 (2.33) | 11.57 (3.55) | 0.807 |
| Diarrhea(%) | 14.0 (56.0) | 13.0 (52.0) | 1 | AST(U/L) | 24.92 (18.93) | 23.92 (10.11) | 0.817 |
| Dyspnea(%) | 8.0 (32.0) | 10.0 (40.0) | 0.768 | ALT(U/L) | 30.64 (50.76) | 24.76 (23.64) | 0.602 |
| Chest pain(%) | 12.0 (48.0) | 6.0 (24.0) | 0.141 | Total bill(mmol/L) | 0.64 (0.37) | 0.59 (0.56) | 0.688 |
| Symptom counts | 5.0 (2.61) | 5.04 (2.59) | 0.957 | Albumin(g/dL) | 4.12 (0.27) | 4.12 (0.31) | 0.965 |
| **Comorbidities** |  |  |  | Platelet(×10^9^ /L) | 265.84 (90.39) | 259.0 (64.67) | 0.76 |
| Any Past History(%) | 4.0 (16.0) | 3.0 (12.0) | 1 | LDH(U/L) | 238.52 (56.91) | 251.92 (120.92) | 0.618 |
| Hypertension(%) | 3.0 (12.0) | 2.0 (8.0) | 1 | Total cholesterol(mg/dL) | 159.56 (32.32) | 160.32 (26.24) | 0.928 |
| Diabetes mellitus(%) | 0.0 (0.0) | 0.0 (0.0) | 1 | HDL(mg/dL) | 46.08 (8.35) | 45.14 (10.16) | 0.725 |
| Dyslipidemia(%) | 1.0 (4.0) | 1.0 (4.0) | 1 | TG(mg/dL) | 133.76 (59.02) | 146.08 (52.5) | 0.439 |
| Thyroid(%) | 0.0 (0.0) | 2.0 (8.0) | 0.47 | Glucose(mg/dL) | 95.76 (25.09) | 94.64 (36.01) | 0.899 |
|  |  |  |  | CRP(mg/dL) | 0.48 (0.59) | 0.56 (1.14) | 0.756 |

BMI: body mass index; HQ: hydroxychloroquine; WBC:White blood cells; LYM:Lymphocytes; RBC:Red blood cells; AST: Aspartate aminotransferase; ALT: alanine aminotransferase; LDH: lactate dehydrogenase; BUN: blood urea nitrogen; HDL: high-density lipoproteins; PT (INR): prothrombin time (international normalized ratio); CRP: c-reactive protein; TG: triacyl-glyceride; SD: standard deviation. *Cardiovascular disease : coronary artery disease, Heart Failure, Arrhythmia

Continuous characteristics are shown as means and standard deviations in brackets, while categorical binary characteristics are shown as counts and percentages in brackets. P-values of continuous variables are based on t-test and p-values of categorical variables are based on chi-square test.
