# Supplementary material for: Use of combined treatment of 3rd-generation cephalosporin, azithromycin and antiviral agents on moderate SARs-CoV-2 patients in South Korea: A retrospective cohort study
Source: PLoS One. 2022 May 4;17(5):e0267645. doi: 10.1371/journal.pone.0267645 (PMC9067652; doi:10.1371/journal.pone.0267645)
Supplement: S2 Table — (DOCX) [file pone.0267645.s005.docx]

**Supplementary Table 2. Clinical outcomes of CA/LoP and CA/HQ groups after propensity score matching**

| **After matching** | **CA/LoP** | **CA/HQ** | **P-value** |
| --- | --- | --- | --- |
| **Number of patients** | 25 | 25 |  |
| **Clinical outcomes** |  |  |  |
| Transfer to tertiary Hospital(%) | 3.0 (12.0) | 1.0 (4.0) | 0.602 |
| Viral clearance(days) | 24.88 (8.81) | 22.92 (8.34) | 0.423 |
| Hospital stay(days) | 22.2 (8.26) | 21.68 (8.24) | 0.825 |
| Symptom resolution(days) | 14.68 (12.15) | 15.92 (11.17) | 0.709 |
