# Supplementary material for: Use of combined treatment of 3rd-generation cephalosporin, azithromycin and antiviral agents on moderate SARs-CoV-2 patients in South Korea: A retrospective cohort study
Source: PLoS One. 2022 May 4;17(5):e0267645. doi: 10.1371/journal.pone.0267645 (PMC9067652; doi:10.1371/journal.pone.0267645)
Supplement: S3 Table — (DOCX) [file pone.0267645.s006.docx]

**Supplementary Table 3. Adverse effects and drug switch percentage after propensity score matching**

| **After matching** | **Standard** | **CA/LoP** | **P-value** | **Standard** | **CA/HQ** | **P-value** |
| --- | --- | --- | --- | --- | --- | --- |
| **Number of patients** | 25 | 25 |  | 15 | 15 |  |
| **Adverse reactions** |  |  |  |  |  |  |
| Nausea & Vomiting(%) | 0.0 (0.0) | 5.0 (20.0) | 0.059 | 0.0 (0.0) | 0.0 (0.0) | 1 |
| Diarrhea(%) | 9.0 (36.0) | 11.0 (44.0) | 0.773 | 4.0 (26.67) | 5.0 (33.33) | 1 |
| Cardiac diseases^*^(%) | 0.0 (0.0) | 0.0 (0.0) | 1 | 0.0 (0.0) | 0.0 (0.0) | 1 |
| Psychological symptoms | 0.0 (0.0) | 1.0 (4.0) | 1 | 0.0 (0.0) | 0.0 (0.0) | 1 |
| Increased AST(%) | 4.0 (16.0) | 4.0 (16.0) | 1 | 3.0 (20.0) | 4.0 (26.67) | 1 |
| Increased ALT(%) | 7.0 (28.0) | 4.0 (16.0) | 0.495 | 3.0 (20.0) | 4.0 (26.67) | 1 |
| Increased Total Bilirubin(%) | 0.0 (0.0) | 3.0 (12.0) | 0.234 | 0.0 (0.0) | 0.0 (0.0) | 1 |
| Increased Cr(%) | 2.0 (8.0) | 1.0 (4.0) | 1 | 0.0 (0.0) | 0.0 (0.0) | 1 |
| Increased BUN(%) | 1.0 (4.0) | 0.0 (0.0) | 1 | 0.0 (0.0) | 0.0 (0.0) | 1 |
| Increased LDH(%) | 18.0 (72.0) | 19.0 (76.0) | 1 | 12.0 (80.0) | 10.0(66.67) | 0.68 |
| Increased CRP(%) | 3.0 (12.0) | 6.0 (24.0) | 0.462 | 1.0 (6.67) | 2.0 (13.33) | 1 |
| **Drug switch** |  |  |  |  |  |  |
| Switch from LoP/R to HQ(%) | 0.0 (0.0) | 0.0 (0.0) | 1 | 0.0 (0.0) | 1.0 (6.67) | 1 |
| Switch from HQ to LoP/R(%) | 0.0 (0.0) | 3.0 (12.0) | 0.234 | 0.0 (0.0) | 0.0 (0.0) | 1 |
| **O2 supply application(%)** | 0.0 (0.0) | 0.0 (0.0) | 1.0 | 0.0 (0.0) | 0.0 (0.0) | 1 |
| **Duration of medication use** |  |  |  |  |  |  |
| Cefixime use(days) | 0.0 (0.0) | 9.2 (3.37) | <0.001 | 0.0 (0.0) | 8.55 (3.5) | <0.001 |
| AZ use(days) | 0.0 (0.0) | 3.88 (1.45) | <0.001 | 0.0 (0.0) | 2.8 (1.08) | <0.001 |
| LoP/R use(days) | 0.0 (0.0) | 8.4 (2.72) | <0.001 | 0.0 (0.0) | 0.07 (0.26) | 0.326 |
| HQ use(days) | 0.0 (0.0) | 0.48 (1.33) | 0.077 | 0.0 (0.0) | 8.73 (2.61) | <0.001 |
| Timing of medication(days)^**^ | 0.0 (0.0) | 13.6 (11.5) | <0.001 | 0.0 (0.0) | 15.0 (10.04) | <0.001 |

^*^1 patient for cardiomegaly in CA/LOP group, 1 patient for tachycardia in CA/HQ group. P-value^a^, p-value^b^, and p-value^c^ respectively represent test results of Standard vs CA/LoP groups, Standard vs CA/HQ groups, and CA/LoP vs CA/HQ groups. P-values of continuous variables are based on t-test and p-values of categorical variables are based on chi-square test. ^**^Timing of medication use is determined by subtracting the start date of medication to the start date of facility operation, February 28^th^, 2020.
