# Supplementary material for: Use of combined treatment of 3rd-generation cephalosporin, azithromycin and antiviral agents on moderate SARs-CoV-2 patients in South Korea: A retrospective cohort study
Source: PLoS One. 2022 May 4;17(5):e0267645. doi: 10.1371/journal.pone.0267645 (PMC9067652; doi:10.1371/journal.pone.0267645)
Supplement: S4 Table — (DOCX) [file pone.0267645.s007.docx]

**Supplementary Table 4. Adverse effects and drug switch percentage of CA/LoP and CA/HQ groups after propensity score matching**

| **After matching** | **CA/LoP** | **CA/HQ** | **P-value** |
| --- | --- | --- | --- |
| **Number of patients** | 25 | 25 |  |
| **Adverse reactions** |  |  |  |
| Nausea and Vomiting(%) | 5.0 (20.0) | 1.0 (4.0) | 0.192 |
| Diarrhea(%) | 12.0 (48.0) | 8.0 (32.0) | 0.386 |
| Cardiac diseases^*^(%) | 0.0 (0.0) | 1.0 (4.0) | 1 |
| Psychological symptoms | 1.0 (4.0) | 0.0 (0.0) | 1 |
| Increased AST(%) | 2.0 (8.0) | 5.0 (20.0) | 0.415 |
| Increased ALT(%) | 3.0 (12.0) | 6.0 (24.0) | 0.462 |
| Increased Total Bilirubin(%) | 1.0 (4.0) | 1.0 (4.0) | 1 |
| Increased Cr(%) | 1.0 (4.0) | 0.0 (0.0) | 1 |
| Increased BUN(%) | 0.0 (0.0) | 0.0 (0.0) | 1 |
| Increased LDH(%) | 20.0 (80.0) | 17.0 (68.0) | 0.519 |
| Increased CRP(%) | 7.0 (28.0) | 7.0 (28.0) | 1 |
| **Drug switch** |  |  |  |
| Switch from LoP/R to HQ(%) | 0.0 (0.0) | 2.0 (8.0) | 0.47 |
| Switch from HQ to LoP/R(%) | 3.0 (12.0) | 0.0 (0.0) | 0.234 |
| **O2 supply application(%)** | 2.0 (8.0) | 0.0 (0.0) | 0.47 |
| **Duration of medication use** |  |  |  |
| Cefixime use(days) | 9.47 (3.21) | 8.45 (3.14) | 0.261 |
| AZ use(days) | 4.04 (1.31) | 2.8 (0.91) | <0.001 |
| LoP/R use(days) | 8.29 (2.63) | 0.24 (0.72) | <0.001 |
| HQ use(days) | 0.48 (1.33) | 8.44 (2.28) | <0.001 |
| Timing of medication(days)^**^ | 11.92 (10.7) | 12.28 (9.32) | 0.9 |

^*^1 patient for cardiomegaly in CA/LOP group, 1 patient for tachycardia in CA/HQ group. P-value^a^, p-value^b^, and p-value^c^ respectively represent test results of Standard vs CA/LoP groups, Standard vs CA/HQ groups, and CA/LoP vs CA/HQ groups. P-values of continuous variables are based on t-test and p-values of categorical variables are based on chi-square test. ^**^Timing of medication use is determined by subtracting the start date of medication to the start date of facility operation, February 28^th^, 2020.
