# Supplementary material for: Use of combined treatment of 3rd-generation cephalosporin, azithromycin and antiviral agents on moderate SARs-CoV-2 patients in South Korea: A retrospective cohort study
Source: PLoS One. 2022 May 4;17(5):e0267645. doi: 10.1371/journal.pone.0267645 (PMC9067652; doi:10.1371/journal.pone.0267645)
Supplement: S5 Table — CA/LoP group is used as the reference. (DOCX) [file pone.0267645.s008.docx]

**Supplementary Table 5. Comparison between CA/HQ and CA/LoP groups in terms of time to symptom resolution, time to viral clearance, and hospital stay duration in crude analysis, multivariable analysis, and propensity score matching analysis. CA/LoP group is used as the reference.**

|  | **CA/LoP vs CA/HQ** | |
| --- | --- | --- |
|  | **Hazard Ratio (95% CI)** | **P-value** |
| **Time to symptom resolution** |  |  |
| Crude, unadjusted cox regression | 1.634 (0.993-2.688) | 0.053 |
| Multivariable Cox regression^*^ | 1.028 (0.563-1.876) | 0.929 |
| Propensity-score analyses |  |  |
| With matching^**^ | 1.090 (0.609-1.953) | 0.771 |
| IPTW^***^ | 1.247 (0.759-2.048) | 0.383 |
| Adjusted for propensity score^****^ | 1.039 (0.562-1.916) | 0.904 |
| **Time to viral clearance** |  |  |
| Crude, unadjusted cox regression | 1.788(1.092-2.924) | 0.021 |
| Multivariable Cox regression^*^ | 1.671(0.919-3.04) | 0.092 |
| Propensity-score analyses |  |  |
| With matching^**^ | 1.452(0.813-2.591) | 0.208 |
| IPTW^***^ | 1.575 (0.939-2.642) | 0.085 |
| Adjusted for propensity score^****^ | 1.699(0.929-3.106) | 0.085 |
| **Hospital stay duration** |  |  |
| Crude, unadjusted cox regression | 1.550 (0.949-2.532) | 0.08 |
| Multivariable Cox regression^*^ | 1.239 (0.689-2.227) | 0.475 |
| Propensity-score analyses |  |  |
| With matching^**^ | 1.233 (0.692-2.193) | 0.477 |
| IPTW^***^ | 1.335 (0.815-2.188) | 0.251 |
| Adjusted for propensity score^****^ | 1.244 (0.69-2.242) | 0.467 |

^*^Hazard ratio from the multivariable Cox proportional hazards model where sex, age, BMI, diagnoses of past history, current use of other medications, level of severity, systolic BP, Diastolic BP, heart rate, white blood cells, initial lymphocyte count, platelet, LDH, and CRP at admission were the covariates. ^**^Hazard ratio from a multivariable Cox proportional hazards model with the same covariates on the matched data set. ^***^Hazard ratio from a Cox proportional hazards model with inverse probability of treatment weighting (IPTW). ^****^Hazard ratio from a multivariable Cox proportional hazards model with the propensity score as an additional covariate.
